# Functionalized Stress Component onto Bio-template as a Pathway of Cytocompatibility

**DOI:** 10.1038/srep35425

**Published:** 2016-10-19

**Authors:** Meysam Keshavarz, Bo Tan, Krishnan Venkatakrishnan

**Affiliations:** 1 Micro/Nanofabrication Laboratory, Department of Aerospace Engineering, Ryerson University, 350 Victoria Street, Toronto, ON, M5B 2K3, Canada; 2 Ultrashort laser nanomanufacturing research facility, Department of Mechanical and Industrial Engineering, Ryerson University, 350 Victoria Street, Toronto, ON M5B 2K3, Canada; 3 Keenan Research Centre for Biomedical Science, St. Michael’s Hospital, Toronto, Ontario M5B 1W8, Canada

## Abstract

This *in-vitro* study introduces residual stress as a third dimension of cell stimulus to modulate the interaction between cells and bio-template, without the addition of either chemical or physical stimuli onto the bio-template surface. Ultrashort Pulsed Laser (USPL) irradiation of silicon-based bio-template causes recrystallization of silicon, which mismatches the original crystal orientation of the virgin silicon. Consequently, subsurface Induced Residual Stress (IRS) is generated. The IRS components demonstrated a strong cytocompatibility, whereas the peripheral of IRS, which is the interface between the IRS component and the virgin silicon surface, a significant directional cell alignment was observed. Fibroblast cells shown to be more sensitive to the stress component than Hela cancer cells. It revealed that cytocompatibility in terms of cell migration and directional cell alignment is directly proportional to the level of the IRS component. Higher stress level results in more cell alignment and border migration width. There is a stress threshold below which the stress component completely loses the functionality. These results pointed to a functionalized bio-template with tunable cytocompatibility. This study may lead to a new tool for the designing and engineering of bio-template.

There is an ample amount of literature surrounding the needs for modulation of cell behavior for bio-applications, which indicates that study of cell interaction with biomaterials/bio-templates is of great importance for controlling cell behaviour, growth, and migration. With advancement in Nano and Micro-scale technologies, a variety of approaches have been developed to engineer *in-vitro* cell interaction, including topographical patterning, changing surface chemistry, mechanical loading (stiffness), and the combinations of these methods^1^. Micro and nano topographic patterns is a popular tool for directional migration of cells. This method mechanically guide the cell migration by restricting cell mobility in channels and grooves^1^,^2^,^3^,^4^,^5^,^6^. Lithography, for instance, is one of the methods to create micro channels with an acceptable feature resolution^7^. The major challenge of this approach is that the fabricated topographic pattern do not represent the true extra cell matrix in nature. Cells in a real tissue are subject to an environment of three-dimensional space, multiple soluble factors, and cell-cell interactions^1^,^8^,^9^. Some effort have been made to partially address this issue. For example, *Zhou et al.* has demonstrated a two-level topographic pattern made by an integrated lithography to cue cells^8^. Modification of surface chemistry is another approach of bio-template. Premnath *et al*. has recently performed an in-depth study of programing cancer cells through phase-functionalized silicon-based biomaterials^10^. It revealed that changing the surface chemistry of the silicon substrate by formation of oxides contributes to the control of directional migration, cytoskeleton shape, and cell population^10^,^11^,^12^. This finding has provided valuable insight into cellular responses to changes of material-phase stimuli. However, the functionality of this method relies on the changing of surface chemistry. Recent studies have shown that mechanical loading (stress and strain) can be implemented as a cue for cell signaling and migration^13^,^14^,^15^,^16^,^17^. However, the application of this method is restricted to polymer-based bio-templates^17^. It cannot be applied to silicon-based bio-templates which are widely used as biocompatible templates, due to the brittle nature of silicon^18^,^19^.

In this research work, we introduced a novel concept of using laser induced residual stress (IRS) on a monocrystalline silicon substrate as a stimuli for cell modulation. This method does not employ chemicals or topographic structures. It stimulates cell migration and alignment through subsurface stress. To the best of our knowledge, this the first time subsurface stress has been used on silicon bio-template for the modification of cytocampatibility. A monocrystalline silicon wafer has been chosen as an experimental substrate because silicon crystals are sensitive to mechanical and thermal excitation. Silicon is increasingly being considered for bio-applications^20^,^21^,^22^. Aside from biological in favor of using silicon as a bio-template, slight changes in crystal orientation of a silicon substrate leads to the build-up of a residual stress component. This unique property can be attributed to the mono crystallinity of silicon r, making it a favourable material for use as a bio-template. In order to accurately tune the functionalization of stress components on the IRS, the bio-template was exposed to the irradiation from an Ultrashort Pulsed Laser (USPL). The combination of shockwave formations and intense thermal expansion generated by the laser irradiation, induces crystal distortion. The resulting mismatch of distorted crystal orientation and virgin crystals causes residual stress. The functionality of the stress component was controlled by varying repetition rate (rep.rate) of the laser. The repetition rate determines the level of stress of the functionalized stress component, and it in turn tunes the degree of biocompatibility of the bio-template. This functionalized property elicits precise control on cell behaviour, such as directional cell migration and self-cytocompatibility. Fibroblast and cancerous (HeLa) cells seeded respectively onto IRS zones and the responses were observed after 24 hours of incubation. Both cell lines showed a significant collective migration tendency towards virgin areas on the bio-template. In contrast to the cell migration trend, cell mobility was confined by the functionalized stress component. In this case, the migration distance can be regulated by the level of stress within the functionalized stress component. The Fibroblast (NIH3T3) cells is found to be distinctively more sensitive to the functionalized stress component. Precisely speaking, the threshold of cytocompatibility is comparatively lower in Fibroblast (NIH3T3) cells. Moreover, directional cell alignment was observed on the proximity of IRS. The results showed that, for the first time, significant promise of creating self-cytocompatibility properties in silicon bio-component without resorting to chemical stimuli and/or topographical structures. Figure 1 is a schematic drawing shows the response of cells to IRS. The cell interactions, topographical, elemental, compositional, and crystallographic properties of the IRS bio-template were characterized by the Scanning Electron Microscopy (SEM), Florescent Microscope, X-ray Diffraction (XRD), Energy Dispersive X-ray (EDX), and Electron Backscatter Diffraction (EBSD), respectively. Finally, to identify the threshold of cytocompatibility, the minimum level of stress to observe cytocompatbility, was evaluated with Micro-Raman spectrum shift.

## Results and Discussion

### Fabrication and characterization of IRS onto bio-template

Interaction of ultra-short pulsed laser with matter falls into two steps: a localized thermal accumulation and shockwave caused by multi-photon absorption, and a blast of plasma formation^23^,^24^. Thereby the sequential interaction of USPL trains with the substrate is triggered to increase the localized functional stress component along with the laser path^25^,^26^. Laser irradiation of silicon-based bio-templates using different laser repetition rates has prompted a gradient of residual stress at the peripheral of IRS^27^,^28^. Amplitude of this laser-induced stressed zone is proportional to the repetition rate of the laser. However for a given pulse width, the gradient of laser fluence decreases with increasing laser pulse repetition rate from 4 MHz to 26 MHz; here, the transmitted energy, in terms of heat and pressure of the shockwave, is intensified. To expound upon the relationship of laser ablation threshold and laser fluence with repetition rate, the following equations were considered;

Laser fluence is given by,


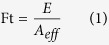


where E is laser pulse energy [J] and A_eff_ is effective focal spot area [cm^2^]. Laser Pulse energy is given by,


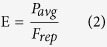


where P_avg_ is the Laser average power [W] and F_rep_ [KHz]. Ablation threshold for a single laser pulse is given by,


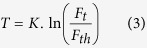


where F_t_ is the laser fluence, F_th_ is the ablation threshold, and K is related both to the absorption depth of the light in the bulk material, and the thermal diffusion length (2√*D* × *τ* where D is the thermal diffusivity and τ is the laser pulse duration).

**Figure 2:**
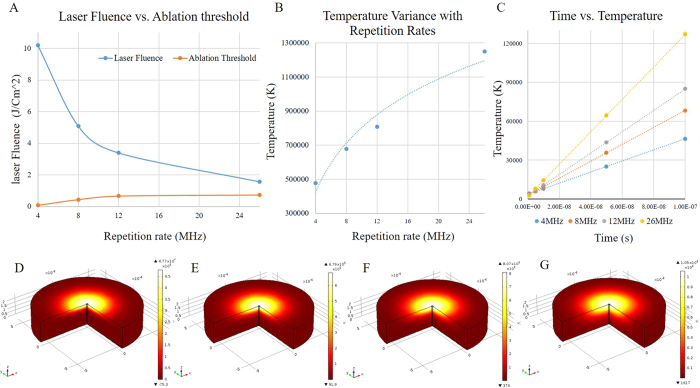
Plot **A**, shows variation of laser fluence versus ablation threshold of Silicon bio-template a distinct laser pulse width of (214 fs) and repetition rates ranging from 4 to 26 MHz. Plot **B**, Temperature variation with rep.rate, shows temperature rise exponentially by increasing laser rep.rate. Plot **C**, shows the temperature vs. heat dissipation time. **D** to **G** show the temperature gradient simulated by COMSOL Multiphysics. (All the data were taken after 10 consecutive pulses).

Figure 2A shows the variation of laser fluence versus the ablation threshold in terms of energy generated per unit area at distinct repetition rates and 214 (fs) laser pulse width. In this plot the effect of pulse-to-pulse interval was not taken into consideration. However, multi-photon absorption simulation using COMSOL Multiphysics unveiled that the accumulative influence of laser pulses increases by decreasing pulse-to-pulse interval, that is, laser rep.rate. When the bio-template is irradiated with a USPL the surface temperature of substrate exponentially rises by increasing laser rep.rate. The difference in temperature between face and subsurface of the silicon bio-template, caused by rapid heat dissipation, creates intricate crystal distortions. This changes the crystal orientation of the IRS zone from inherent (100) to (211) and a different order of crystallinity. Another advantage of utilizing tunable USPL is the ability to precisely modulate the IRS, avoiding collateral damage created by exceeding the ablation threshold of an irradiated bio-template. In particular, amplifying the repetition rate from 4 MHz to 26 MHz causes extensive accumulation of heat on the exposure zone - owing to maximizing laser matter interaction time, more thermal and shockwave energy is transferred to the substrate. Therefore, incremental heat generation intensifies thermal stress, resulting in formation of the laser induced residual stress zone. Earlier mentioned laser parameters such as fluence and rep.rate also have a similar effect in amplifying the laser shockwave.

In an attempt to understand the heat accumulation generated by the interaction between an ultrafast laser and a bio-template, COMSOL Multiphysics was exploited to simulate the average heat flux disturbed during multiphoton absorption. As mentioned, the simulation was created on COMSOL Multiphysics software and the built-in Heat Transfer in Solids Module was used to solve the following heat transfer equation:


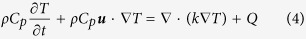


where k is the thermal conductivity of silicon, ρ is the material density, and Cp is the heat capacity of silicon substrate at constant pressure. The pulse width and rep.rate of the USPL were varied for each computational run of the simulation by adjusting the parameters for each new computation. To represent the silicon wafer, a cylindrical geometry was created with a thickness of 1 μm and with a radius identical to the spot size (5 μm) in order to facilitate more precise meshing. A Gaussian pulse (I) was used to mimic the distribution of intensity within a laser beam, the highest intensity being in the center with decreasing intensity towards the circumference. A standard deviation of r_spot/3, where r_spot is the radius of the circular spot, was entered to create increasingly higher temperatures in the centre of the ablation spot. This allowed the simulation of a more realistic temperature distribution. Within the heat flux equations of COMSOL, the laser power (p_laser) was multiplied to the Gaussian functions to produce a gradient intensity (hf) within the laser spot area:


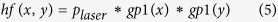


The rep.rate and pulse width were incorporated into a single equation to create a rectangular (rect2) pulse train (an3) with parameters that could be manipulated:


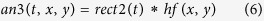


A heat flux function (an4) was then formulated:


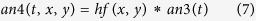


Finally, within the Heat Transfer in Solids Module, the above equation was entered as a general inward heat flux to produce:


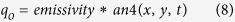


Here, emissivity was taken as 0.8. Within COMSOL, the bottom surface of the silicon wafer geometry was thermally insulated and the initial temperature of the wafer was defined at 293.15 K. A free tetrahedral mesh was used on the entirety of the geometry with a maximum element edge length of 1.8E-6 meters. Upon completion of the computations, the wafer thickness was scaled up to five times to allow improved observation of the depth of the heat gradient within the thickness.

For the following study, a classic heat transfer model was applied in order to describe the phenomenon of accumulative temperature rise caused by amplifying the repetition rate from 4 MHz to 26 MHz. However, as calculated and plotted on Fig. 2A, laser fluence is expected to decrease with increasing repetition rates. Also, shortening pulse to pulse interval at higher repetition rates, increases the initial temperature for the subsequent pulse. Therefore, heat accumulation is expedited by multiplying the number of pulses, as shown on Fig. 2B. As seen in Fig. 2C, heat dissipation time is longer than pulse interval at a higher rep.rate and thus, thermal energy would be expected to build up by increasing the rep.rate. In contrast, at a lower rep.rate, accumulated thermal energy is insufficient, since the heat induced by a single pulse has dissipated by the time the next pulse arrives. Figure 2D to G show the heat generated by multiphoton absorption at different rep.rates, the cross sectional view displays the heat dissipation depth and expansion of IRS at higher rep.rates. The pulse-to-pulse duration is a function of the repetition rate of a laser and therefore, has a significant influence on laser matter interaction - the substrate in the focal point gains energy and is thermally loaded by sequential pulses. Therefore, different repetition rates (4, 8, 12 and 26 MHz) at constant a 214 fs pulse-width have been implemented to irradiate the bio-template, through which the functionalized stress component can be induced. Moreover, to characterize the formation of distinct crystal orientations XRD and EBSD have been carried out.

**Figure 3:**
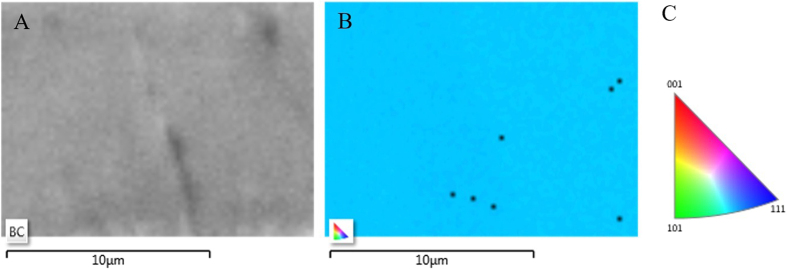
(**A**) Back scatter scanning area. (**B**) EBSD mapping of the IRS zone. (**C**) EBSD relative Euler orientation map. (The color is related to the crystallographic direction that is parallel to the surface).

The crystalline orientation map of the IRS zone is shown in Fig. 3. The mapping area shown in Fig. 3A is 200 μm^2^, with a mapping step of 0.2 μm where an index of over 85% was achieved. The different colors of the orientation map correspond to the different crystallographic orientations of scanned area. The (001) crystallographic plane was set as the reference direction. Hence, comparison of collected information from backscattered Kikuchi diffraction patterns at each EBSD mapping point revealed a deviation from the reference orientation. The EBSD mapping result shows that the IRS zone is blue in color, whereas crystal orientation on the unirradiated zone is red in color (according to the EBSD relative Euler orientation map Fig. 3C) which indicates a deviation from (100) to (211). The color variation of the orientation map shown on Fig. 3B corresponds to the different crystallographic orientations. In this case, (211) is the dominant crystal plane. The corresponding orientation for each color is represented on Fig. 3C. This EBSD outcome is in line with the resulting XRD pattern.

**Figure 4:**
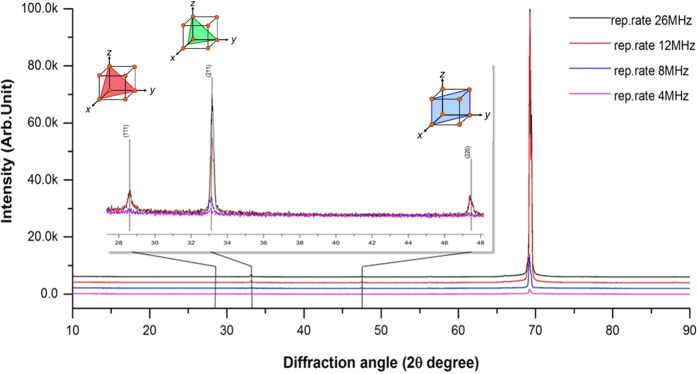
XRD patterns of laser irradiated bio-templates at 4, 8, 12, and 26 MHz. Enlarged pattern shows emerging peaks, representing of (111), (211) and (221) crystal orientations.

**Figure 5:**
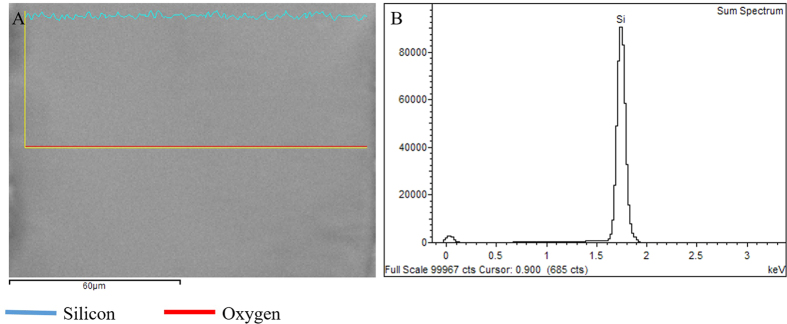
EXD spectrum through LIRS IRS and plain bio-template. (**A**) SEM image of take spectrum. (**B**) EDX Spectrum identifying the presence of Oxygen.

The influence of laser irradiation on the crystal orientation of bio-templates was acquired by utilizing X-ray diffraction, as plotted on Fig. 4. Diffraction patterns of the IRS bio-templates at different laser repetition rates were compared with a single crystal silicon substrate as a reference. D-spacing calculation of diffraction pattern of reference substrate shows a characteristic peak at 2θ (69.2), which stands for (100). While after exposure to an USPL, distinct peaks such as (111), (211) and (220) were emerged. An increment in intensity of a peak represents (211) crystal orientation observed by increasing repetition rate of the laser pulse from 4 to 26 MHz. However, this peak has relatively lower intensity compared to a dominant (100) peak due to the fact that only a thin facial layer of a bio-template undergoes crystal orientation changes. The XRD result agrees with the EBSD outcome, both of methods demonstrating changes of crystal orientation on IRS zones. Further characterization has been done using EDX to examine the possibility of surface oxidation or surface compositional changes. The XRD analysis revealed that the compositional aspect of all samples remained intact. Surface compositional characterization of IRS at different laser repetition rates was examined by EDX. Figure 5 shows an EDX spectral. Elemental analysis of the spectrum revealed that there is no compositional difference in comparison to a plain silicon wafer. This indicates that multiphoton substrate interaction to induce functional stress component using the previously mentioned parameters, does not cause compositional changes. However, alternations in crystal orientation have clearly been observed in XRD patterns and through EBSD mapping.

**Figure 6:**
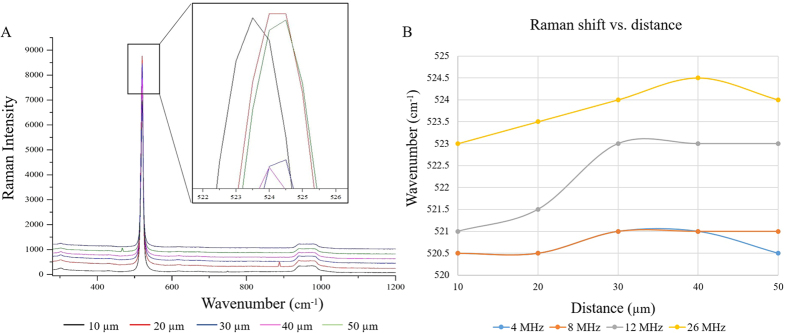
(**A**) Raman spectra of IRS at 26 MHz. (**B**) Raman shifts at 4, 8, 12, and 26 MHz.

To analyze the relationship between laser irradiation energy and functionalized stress components, IRS was characterized by Raman spectroscopy in terms of wavelength shift. In general, as a result of compressive stress, Raman frequency increases while tensile stress results in downward shift of Raman frequency^29^,^30^,^31^,^32^. Figure 6A shows Raman spectra of a bio-template irradiated at 26 MHz. As shown in Fig. 6A, there is a slight difference in measured peak position when moving outward within the IRS zone. Raman shift variation measured at each 10 μm (up to 50 μm) from the proximity of IRS at different rep.rates, are illustrated in Fig. 6B. The characteristic Raman scattering frequency of the silicon-based bio-template used in this study was ω = 521 cm^–1^ however, this value shifted as a result of USPL-induced stress. Mapping of Raman shift differences through the IRS zone leads us to determine the thresholds of HeLa and Fibroblast (NIH3T3) cell lines in sensing the minimum required functionalized stress component by which a cell-free zone can be created as a result of collective cell migration. Slight changes in measured frequency, in this case for 0.5 cm^−1^ peak shift in silicon, represents a stress sensitivity of about 4 KPa^32^. The relation between Raman peak shift and stress was assumed to be simply linear, and the effect of all stain tensor components are not considered^31^,^32^. Components of strain in crystalline materials affect the frequency of the Raman phonons and as such, the position of the Raman peak. For instance, for uniaxial or biaxial stress in the (100) plane of silicon, this relation is:


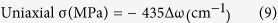



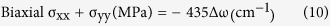


**Figure 7:**
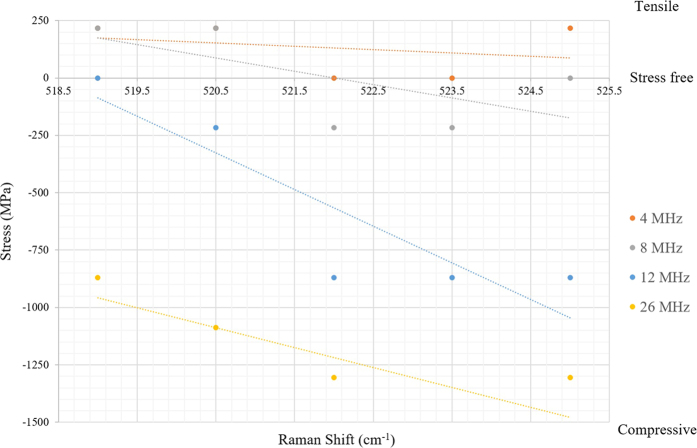
Shows magnitude of stress component as function of repetition rate.

Laser fluence that can be precisely tuned through the adjustment of laser parameters, such as rep.rate, laser power, to accurately determine the tensile and compressive stress. The results from cell culture show that the degree of functionalization of the induced stress component depends on the level of stress. A general observation is that a considerable amount of compressive stress was induced at rep.rates higher than 8 MHz, as showed in Fig. 7. Comparison of collective cell migration distances from the IRS, demonstrated that compressive stress has a profound impact on cell response.

### Cell interaction with IRS bio-template

#### Collective cell migration

**Figure 8:**
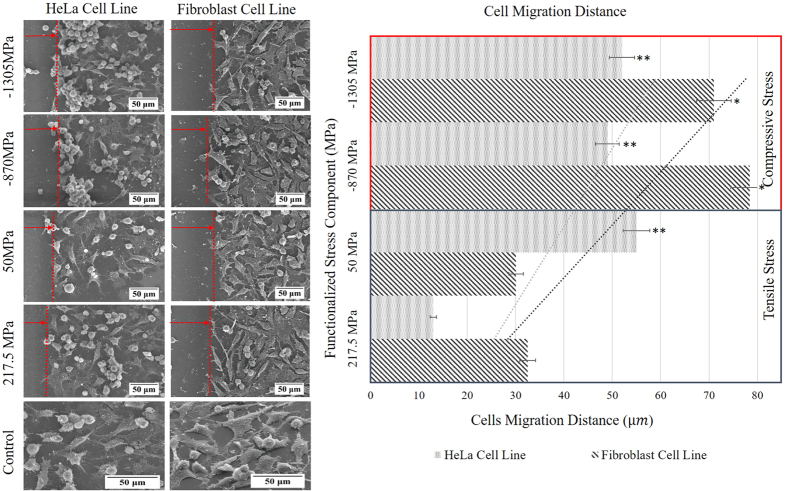
Collective cell migration of HeLa and Fibroblast (NIH3T3) in response to functionalized stress component induced on bio-template at different rep.rate are shown on SEM images. The histogram on the right illustrates distance of cell migration corresponding to the magnitude of functionalized stress component. Error bars show SEM; two independent experiments were repeated with n = 3 in each experiment. Statistical significance is shown with *p < 0.05, **p < 0.01.

We employed a culture medium that contained 3× 10^6 ^cells/mL; HeLa and Fibroblast (NIH3T3) cell lines were separately seeded and incubated for 24 hrs. Interestingly, collective cell migration from the IRS zone towards the plain surface was observed on fluorescent and SEM images. As shown on images in Fig. 8 both Fibroblasts (NIH3T3) (Fib) and cancerous cells (Hela) responded to a functionalized stress component as a migration stimulus. Thus the sensitivity of cells to the residual stress required to cue them has been secured as a threshold of cytocompatibility. Moreover, as evidenced in Fig. 8, higher sensitivity of cells to the compressive stress compared to the tensile stress is significantly noticeable. As presented in Fig. 8 (cell migration distance) by intensifying the repetition rate, compressive stress increases and hence the cells migration distance was broadened. It is implied that, as a result of increasing the repetition rate, the intensity of residual stress has magnified in linear regression with the average migration distance of cells from IRS zones. Therefore, distinct boundary on proximity of the IRS zone has created a cell-free zone. Given the cell-free zone indirectly rule out the possibility of topographical changes in terms of surface 20 ness, as the profound influence of surface roughness on proliferative behavior of cell by which increment of roughness enhances the cell growth and proliferation is well known^33^. The width of this cell-free zone is directly proportional to the induced residual stress and hence laser repetition rate. It can be seen that induced residual stress on bio-templates can be functionalized in order to regulate cells behavior.

**Figure 9:**
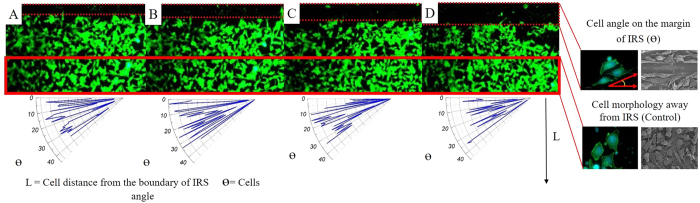
Polar diagrams show, decrease of cell angle by increasing the magnitude of functionalized stress component. (**A**–**D**) The fluorescent images of Fibroblast (NIH3T3) cell lines seeded on the IRS bio-template with different magnitudes of functionalized stress component, 217.5, 50, −870 and −1305 MPa respectively. Comparison of images (**A**,**D**) shows that by increasing the functionalized stress component, diversity of cell angle decreases.

The measured Raman shift indicates that compressive stress is dominant at higher laser repetition rates (Fig. 7). In addition, transition of tensile to compressive stress at 4 and 8 MHz has been determined to be a threshold of cytocompatibility, by which a minimum width of 30 μm for Fibroblast (NIH3T3)and 22.3 μm for HeLa have been formed through collective cell migration. Higher sensitivity of Fibroblast (NIH3T3) cells was noticed compare to HeLa cells. Due to the higher mobility and smaller size of the cytoplasm of HeLa cells, their reduced sensitivity to the IRS can be speculated. Additionally, cytoskeleton breakage on cells moving away from the IRS and protrusion on cells who encounters IRS were observed. Cell response to IRS was characterized in terms of cell directionality and persistence distance. The polar diagrams (Fig. 9) show directionality of collective cells on four different conditions within a 100 μm proximity of central the IRS zone. Given polar diagrams on Fig. 9, a significant difference between cell angles on Fig. 9A,D has been observed, each image compares a tendency of cell alignment on the proximity of the IRS with the control area far from the IRS. To calculate directionality of the cell migration, symmetry of IRS zone was taken into account. The boundary of cell interaction with IRS was defined as (0, 0) of the virtual origin, an absolute value of cell angle considered as directional value. At higher functionalized stress component induced onto the bio-template by shorter laser pulse separation time (high rep.rate), cells tend to be oriented more closely along the X vector of the virtual coordinate than for a longer pulse separation time (short rep.rate). Cell angles gradually increase towards the unirradiated zone on which cells on this zone do not show preferential direction. In other words, cells tend to become parallel at the proximity of IRS. The same propensity was observed on HeLa cell lines. Preferable migration towards plain areas was pronounced on cells at outer edges of peripheral laser induced zones. This attribute allowed us to manipulate the directionality and morphology of the cells. It can be concluded that cell morphology is an effect of cell migration and self-adoption of a cell’s cytoskeleton that implies better understanding of the effects of controlling cell behaviour. Although the morphology of cells away from the IRS zone (control area) showed a tendency toward a polygonal shape.

### Cytocompatibility to induced residual stress (inter-cellular response)

**Figure 10:**
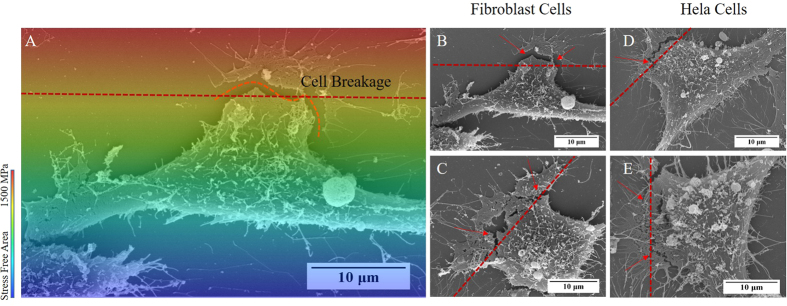
(**A**) Schematic illustration of the distribution of functionalized stress component and cyto-breakage as a result of cell-IRS interaction. (**B**,**C**) SEM images shows cyto-breakage of Fibroblast (NIH3T3) cells. (**D**,**E**) same phenomena has observed on HeLa cells facing IRS as well. (Arrows and dashed line indicate the side of breakage and the boundary of IRS zone respectively).

**Figure 11:**
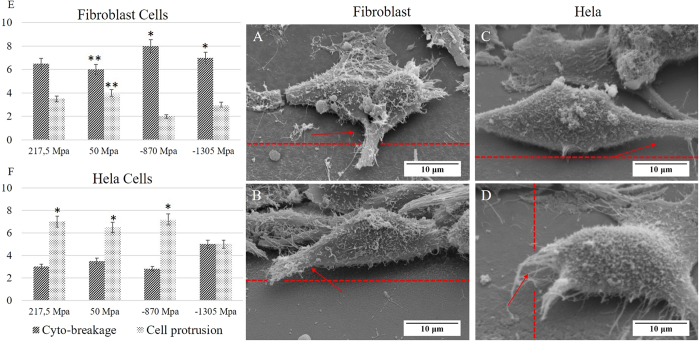
SEM images show cells protrusion on the side facing IRS. (**A**,**B**) Fibroblast (NIH3T3) cell line, and (**C**,**D**) Hela cell lines. The statistical diagrams (**E**,**F**) compares the number of cell protrusions and cyto-breakages on 4, 8, 12 and 26 MHz for Fibroblast (NIH3T3) and HeLa. Error bars show SEM; two independent experiments were repeated with n = 3 in each experiment. Statistical significance is shown with *p < 0.05, **p < 0.01. (Arrows are indication the lamellipodium protrusion on the SEM images and dashed line indicate the boundary of the IRS zone).

Quantitative information about cells-matter interaction can be inferred by investigation of cell morphology^34^,^35^. Therefore, interaction of an individual cell with functionalized stress component was examined. Two distinct phenomena of cyto-breakage and cell protrusion were observed on cells in the proximity of the IRS zone. Upon formation of filopodia after seeding the cells on the bio-template, they perpetually sense their environs to further formation of lamellipodia and ultimately direct cell movement. Cell retraction by which cyto-breakage occurred was reaction to failed adhesion and premature formation of filopodia facing the IRS. The SEM images of HeLa and Fibroblast (NIH3T3) cells, shown in Fig. 10, indicate that although filopodia have been developed on the both sides of cells, cyro-breakages have taken place on the side of the cell facing to the IRS zone. This is evidence that cell migration through retraction and expansion is prohibited by a functional stress component. On the other hand, during 24 hours of incubation, it is likely that cells migrate towards IRS zone. In this case, cells tend to protrude on the side facing the IRS zone as shown in Fig. 11. Additionally, as statistical survey in Fig. 11E,F evidenced that cell protrusion is more dominant on HeLa cell line compared to Fibroblast (NIH3T3) cell line, in which cyto-breakages was common. Lamellipodia are the actual motor which pulls the cell forward during the process of cell migration. Protruding of lamellipodium, indicates no preference of a cell to attach to the IRS zone as SEM images showed on Fig. 11A–D. Therefore, disinterest of lamellipodia to the IRS zone causes cell protrusions. On the other hand, there is a strong belief that the cell crawling in the process of migration is initiated by the development of stable filopodium-substrate adhesion, followed by development of lamellipodium^36^,^37^,^38^. Hence cell protrusion is dependent on collaborative interaction of filopodia and lamellipodia^39^. However lamellipodia function as key structure on cell migration, filopodia extensions are essential for guidance and cellular architecture^40^. Investigation of cell protrusion were made possible by high resolution SEM imaging and tilting technique of substrate up to 45°.

### Filopodial extensions

**Figure 12:**
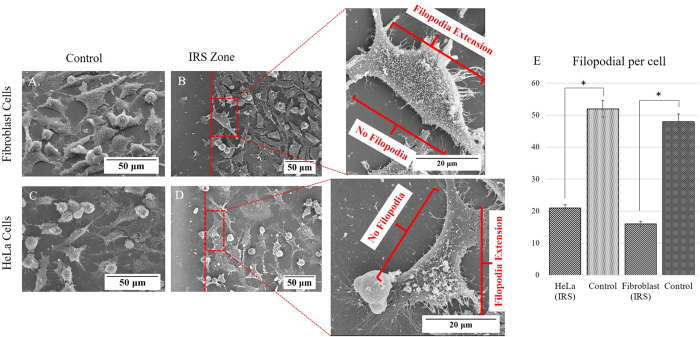
(**B**,**D**) SEM images show the Filopodial extension on the side of the cells opposite to the IRS zone. (**A**,**C**) Illustrate the Fibroblast (NIH3T3) cells and HeLa cells respectively on the control samples. Bar graph on the left side (**E**) comparing the number of filopodia of HeLa and Fibroblat seeded on the IRS bio-template and respective control samples. (Hundred cells were counted for each cell group of samples; error bars denote SD. for each group).

Filopodia serve as probing sensors at the frontier of cells that are extended beyond the leading edge of lamellipodia. This attribution leads them to have a significant function in cell migration as well as cell environmental sensing^37^. The number of filopodial extensions are the evidence of cell preference to adhere and grow^41^. Quantitative image analysis, revealed that the side of cells facing the peripheral of the IRS zone has relatively no or a reduced number of filopodial extensions compared to the opposite side of cell that faces untreated substrate (control area) as depicted in Fig. 12E. This immature development of filopodial extensions was observed on both cell lines of HeLa and Filapodia as shown on Fig. 12. In addition, the formation of new filopodia has proven to be strongly dependant on pre-existing filopodia. Taking into account the attributes of filopodial, the two main functions of environmental sensing and driving force generated for cell migration together with filopodial-originated actin bundles, best describe the role of these needle-like structures in directing cell migration^42^,^43^. Filopodia are the starting point for essential adhesion and movement. Therefore, the final cellular position, actin bundles, and formation of new filopodia are affected by interaction of these structures with IRS.

### Stress fibres and cell migration

**Figure 13:**
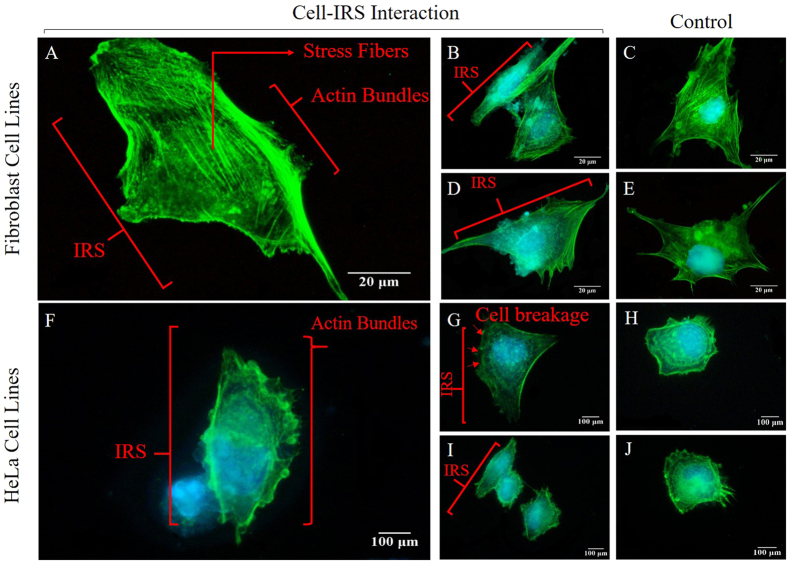
High magnification Fluorescent images (single cell analysis) of Fibroblast (NIH3T3) and HeLa cell lines. (**A**,**F**) Show single Fibroblast (NIH3T3) and HeLa cell interacting with IRS, arrows indicate both sides of a cell. (**B**,**D**) Are example of Fibroblast (NIH3T3) cells seeded on the laser irradiated bio-template at 26 and 4 MHz, compared to (**C**,**E**) where extension of filopodia and lamellipodia are directional. (**G**,**I**) Show the HeLa cells seeded on the laser irradiated bio-template at 4 and 26 MHz respectively (arrows indicate the cell breakage on side of cell facing the IRS). (**H**,**J**) Are HeLa cells on the control area. (Optical images showing actin cytoskeleton (green) and nuclei (blue) of cells after 24 hours of culture on IRS bio-template).

Stress fibers play an important role in providing numerous functions such as cellular contractility, cell adhesion, and migration^44^. Upon attachment of a cell to the bio-template, the pulling force required for traction is provided by adhesion. Thus, cell transition processes are made possible by contraction of the cell body to the new site. Vacillant cells such as Fibroblast (NIH3T3) showed oriented ventral stress fibers perpendicular to the vector of migration. Moreover, a higher concentration of actin bundles formed on the locomotion face of cells are observed. This is caused by stronger adhesion at the migration side compared the rear. Cells in the proximity of IRS have shown, actin bundle concentration on the opposite side of cell breakage, while stress fibers are stretched perpendicular to the direction of migration. (Fig. 13). Taken together, here we reported a novel method of remote stimulation of cell migration by laser-induced residual stress on silicon-based bio-template. It provided a precise manipulation of cell-bio-template interaction, unlike topographical and/or chemical surface modification, cell response to IRS relies on environmental probing of cells.

## Conclusion

In this research work we created an IRS component on a bio-template, which regulates filopodia formation, adhesion, and collective cell migration. Fluorescent and SEM imaging revealed that at the periphery of IRS cell morphology and direction are strongly dependent on the level of stress and the formation and expansion of fiopodia are directly altered by IRS component. It was found that varying laser parameters have a significant influence on the magnitude of stress and consequently the distance of cell migration can be controlled by carefully tuning the laser parameters. Cell culture study indicates that in order for a cell to respond, the induced residual stress must reaches a minimal level, which is defined as threshold of cytocompatibility. The findings of this study opens a new front on cell regulation and cell patterning using silicon based bio-templates.

## Materials and Methods

### Materials and laser irradiation

High-quality polished single crystal N-type silicon wafers with crystallographic orientation of (100) and 625 μm ±25 μm thickness were supplied by University Wafer. After dicing the wafers into 2 cm^2^ square-shape samples, they were ultrasonically cleaned in a 50 degree Celsius acetone bath for 15 minutes, followed by rinsing and drying. In order to induce residual stress, these substrates were exposed to a diode-pumped, Yb-doped femtosecond laser beam (Clark-MXR, Inc.; IMPULSE Series ultrashort pulse laser). This laser system is capable of producing central wavelengths of 1040 nm and a laser pulse width varying from 214 fs to 1428 fs. As well as, the laser pulse repetition rate is tuneable between 4 MHz (Low rep.rate) and 26 MHz (High rep.rate). The maximum operating average power is 16 W. Utilizing an ultrashort pulsed laser (USPL) (femtosecond range) is advantageous since the absorption of multiphoton excitation energy is confined to the localized laser beam spot within the focal point. This results in minimal collateral damage caused by laser-matter interaction. To precisely functionalize the stress component on the IRS, a pulse repetition rate of 4, 8, 12 and 26 MHz, and scanning velocity of 10 mm s^−1^ were implemented. Laser pulse width and average power were set up and remained constant at 214 fs and 16 W respectively. The laser spot-size at the focal point was adjusted to 10 μm diameter after manipulation by a pair of galvanometers through optics lenses. Prior to seeding the cells, the sample was irradiated at the focal point by a computerized Galvano scanner to precisely implement an IRS biomimetic architecture.

### Cell culture and seeding

In order to remove all debris and impurities, samples were ultrasonically cleaned, following the procedure as previously mentioned. Laser treated bio-templates were rinsed with Phosphate Buffered Saline (PBS) (1X strength compose of: 138 mM NaCl, 2.7 mM KCl, 1.9 mM NaH_2_PO_4_, 8.1 mM Na2HPO4) before seeding the cells then dried and sterilized under UV light for 20 minutes. Fibroblasts cell lines (NIH3T3) and human cervical cancer cell lines (HeLa, ATCC, American Type Culture Collection, and ATCC No. CCL-2) were employed in cell experiments to ascertain comparative cell controllability of mammalian and cancer cell lines. Fibroblast (NIH3T3) cells were grown in a DMEM medium containing 10% heat activated fetal bovine serum with 1% penicillin-streptomycin antibiotics (Pen-strep). HeLa cells were grown in a DMEM-F12 medium supplemented with 10% fetal bovine serum and 1% Pen-strep. Subsequently, the cells were separately cultured on the substrates that are placed in petri dishes with seeding density of 750,000 cells/cm^2^ of substrate surface area. The petri dishes were placed in an incubator for 24 hours at 37 °C temperature.

#### Statistics

All cell assay were carried out at least three times and the data points are averages unless otherwise mentioned. The error bars indicate as mean ± standard deviations (SD). Statistical significance were completed using one-way analysis of variance (ANOVA), with *p < 0.05, **p < 0.01 suggesting significant difference. Additionally, the control sample were compared for each significant level shown in (Figs 8 and 11).

### Cell imaging and morphology

The surface morphology of the samples, seeded with cells, were observed using a (Hitachi, SU-1500) scanning electron microscope (SEM). For this regard, after the prescribed time period, spent medium were aspirated. This was followed by the fixing of samples in 2% glutaraldehyde in 0.1 M pH 7.3 sodium cacodylate buffer for an hour. Next, the samples were immersed in 0.1 M sodium cacodylate buffer with 0.2 M pH 7.3 sucrose for 20 minutes. Preceded by dehydration as an increasing concentrations of alcohol for 20 minutes. The samples were then dried to a critical point. At the conclusion of the experiment, cells were ready to be directly observed using Scanning Electron Microscopy (SEM). Images were acquired at 100× to 10,000× magnification with an accelerating voltage of 10 kV, and a probe current of 50 to 60 μA.

### Fluorescence microscopy

To conduct fluorescent visualization the samples are first fixed in methanol-free paraformaldehyde followed by incubation in skim milk to prevent non-specific binding. Actin and cytoskeleton are stained by incubation of the samples with phalloidin-Alexa Fluor 488 (Life Technologies) followed by DAPI (4′,6′-diamidino-2-phenylindole, Life Technologies) to stain the nucleus. The samples are studied using an epi-fluorescent Nikon E-400 microscope (Nikon, Canada).

### Electron backscattered diffraction (EBSD)

A fundamental difference between EBSD and XRD is that X-ray penetrates through the entire thickness of the sample with a 10 mm beam size, whereas EBSD receives backscattered electrons from the top 10–50 nm due to limited backscattering depth of the electrons [22]. The local change in the crystal orientation due to laser strike associated with rapid cooling and shock waves can be determined using EBSD. Crystal orientation was measured with EBSD systems, Oxford Instruments INCA interfaced to a Hitachi SU3500 SEM with a LaB6 electron gun. These systems can automatically obtain a crystal orientation map by scanning a rectangular area on the surface of the specimen, which is tilted 70° from the horizontal.

### X-ray diffraction (XRD)

The elemental composition and crystal structure of the substrate was characterized using Rigaku MiniFlex 600 Benchtop X-ray diffraction (XRD) under Cu-Kα radiation (λ = 0.154 nm). Additionally, XRD analysis of the IRS revealed a pronounced (211) peak that emerged when the laser rep.rate was increased. These spectra are in well agreement with EBSD results in Fig. 5. Secondary phases were not detected in the XRD spectra of IRS samples, which shows there is no compositional deviation from an untreated silicon wafer. To further corroborate with the XRD analysis, Raman and EDX spectra were acquired.

### Energy-dispersive X-ray spectroscopy (EDX)

Further surface analytical techniques were employed to identify possible compositional changes on the IRS zone and compression within the untreated silicon surface. The advantage of employing SEM/EDX lies in shallow penetration depth of the interactive electron beam in contrast to XRD. Thus, surface elemental analysis can be achieved.

### Micro-Raman spectroscopy (μRS)

Micro-Raman spectroscopy is capable of characterizing types of stresses (Tensile/Compressive) within a small measurement spot size, below 5 μm in diameter, and a submicron spatial resolution^30^. Although Micro-Raman spectroscopy was mostly being used in chemical composition studies as a complementary technique to other methods, deducing crystallinity information is advantageous to this technique as a non-destructive method to measure residual stress^29^. Raman spectra were obtained using a Bruker SENTERRA, Dispersive Raman Microscope having a central wavelength of 532 nm as an excitation source. The Raman shift measurements were taken perpendicularly along the IRS of several samples at 10 μm intervals. Through the known relationship between the Raman shift and stress, the stress distributions on the laser-treated samples were determined.

## Additional Information

**How to cite this article**: Keshavarz, M. *et al*. Functionalized Stress Component onto Bio-template as a Pathway of Cytocompatibility. *Sci. Rep.* **6**, 35425; doi: 10.1038/srep35425 (2016).

## Figures and Tables

**Figure 1:**
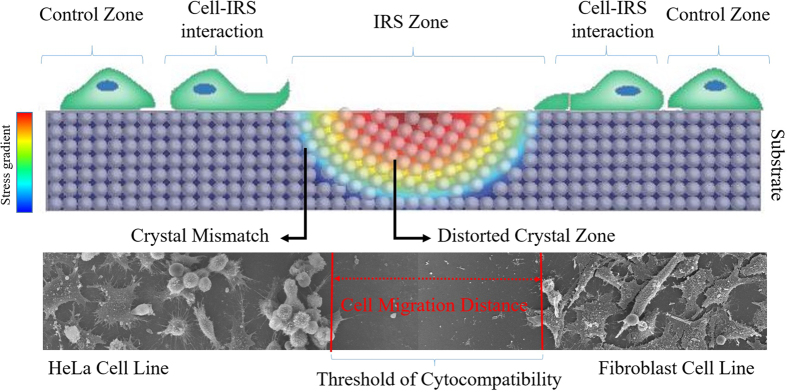
Schematic illustration shows the response of cells to IRS. Two types of encounters on the proximity of IRS are: cell breakage and cell protrusion. SEM images of HeLa and Fibroblast (NIH3T3) represent collective cell migration towards stress-free area on the bio-template.
